# Development of Machine Learning Tools for Predicting Coronary Artery Disease in the Chinese Population

**DOI:** 10.1155/2022/6030254

**Published:** 2022-11-17

**Authors:** Tiexu Zhang, Shengming Huang, Pengfei Xie, Xiaoming Li, Yingxia Pan, Yue Xu, Peng Han, Feifei Ding, Jiangman Zhao, Hui Tang

**Affiliations:** ^1^Department of Cardiovascular Medicine, The First People's Hospital of Pingdingshan, Pingdingshan 467000, China; ^2^Department of Internal Medicine, Luohe Central Hospital, Luohe, 462000 Henan, China; ^3^Health Management Center, Luohe Central Hospital, Luohe 462000, China; ^4^Shanghai Biotecan Pharmaceuticals Co., Ltd, Shanghai 201204, China; ^5^Shanghai Zhangjiang Institute of Medical Innovation, Shanghai 201204, China

## Abstract

**Purpose:**

Coronary artery disease (CAD) is one of the major cardiovascular diseases and the leading cause of death globally. Blood lipid profile is associated with CAD early risk. Therefore, we aim to establish machine learning models utilizing blood lipid profile to predict CAD risk.

**Methods:**

In this study, 193 non-CAD controls and 2001 newly-diagnosed CAD patients (1647 CAD patients who received lipid-lowering therapy and 354 who did not) were recruited. Clinical data and the result of routine blood lipids tests were collected. Moreover, low-density lipoprotein cholesterol (LDL-C) subfractions (LDLC-1 to LDLC-7) were classified and quantified using the Lipoprint system. Six predictive models (k-nearest neighbor classifier (KNN), logistic regression (LR), support vector machine (SVM), decision tree (DT), multilayer perceptron (MLP), and extreme gradient boosting (XGBoost)) were established and evaluated by the confusion matrix, area under the receiver operating characteristic (ROC) curve (AUC), recall (sensitivity), accuracy, precision, and F1 score. The selected features were analyzed and ranked.

**Results:**

While predicting the CAD development risk of the CAD patients without lipid-lowering therapy in the test set, all models obtained AUC values above 0.94, and the accuracy, precision, recall, and F1 score were above 0.84, 0.85, 0.92, and 0.88, respectively. While predicting the CAD development risk of all CAD patients in the test set, all models obtained AUC values above 0.91, and the accuracy, precision, recall, and F1 score were above 0.87, 0.94, 0.87, and 0.92, respectively. Importantly, small dense LDL-C (sdLDL-C) and LDLC-4 play pivotal roles in predicting CAD risk.

**Conclusions:**

In the present study, machine learning tools combining both clinical data and blood lipid profile showed excellent overall predictive power. It suggests that machine learning tools are suitable for predicting the risk of CAD development in the near future.

## 1. Introduction

Coronary artery disease (CAD) is a cardiovascular disease (CVD) which has been found to be the leading cause of mortality worldwide [1] and caused by atherosclerosis, which can be manifested by typical symptoms such as stable angina, unstable angina, myocardial infarction (MI), or sudden cardiac death without any preceding symptoms [2]. So far, coronary angiography is the gold method for CAD diagnosis, but it is an invasive and unpractical for universal screening [3]. Hence, finding cost-effective methods to predict CAD are a major challenge in public health.

Being a complex disease, CAD is caused by genetic and environmental factors as well as the interactions between these factors [4] The well-known risk factors for CAD development include hypertension, dyslipidemia, older age, diabetes mellitus, overweight, and smoking [5, 6]. Furthermore, the prevalence of CAD varies greatly according to the geographical locations, ethnicity, and gender [7].

Besides that, a previous research study indicated that low-density lipoprotein cholesterol (LDL-C) is a primary risk factor for CVD [8], and lowering LDL-C levels with medications have been proved to be effective for primary and secondary prevention [9]. LDL-C is composed of heterogeneous particles with different density and size, which could be classified into two subgroups including small dense LDL-C (sdLDL-C) and large buoyant LDL-C (lbLDL-C) [10]. Moreover, LDL-C was divided into 7 subfractions (LDLC-1 to LDLC-7), of which LDLC-1 and LDLC-2 belong to lbLDL-C, while LDLC-3 to LDLC-7 is defined as sdLDL-C, according to their density and size [10]. Over the past few years, many preventive and therapeutic methods have substantially improved the prognosis of patients with CAD or other CVD [2, 11]. However, the risk of such diseases remains high, and their progression could be halted only in a few patients by using drugs including aspirin, statins, and *β*-blockers [12]. When lowering LDL-C levels to optimal levels, the risk of cardiovascular events still exists [13]. The Atherosclerosis Risk in Communities (ARIC) study showed that sdLDL-C levels may partly account for this residual risk. Since sdLDL-C particles contain less cholesterol and are smaller, increased sdLDL-C levels represent an increase in the amount of atherogenic LDL particles, which LDL-C levels may not represent [14]. In addition, a previous research study has shown that sdLDL-C is considered as an important biomarker for predicting CVD [15]. It is reported that sdLDL-C has stronger transfer ability moving from the vessel lumen into the subintimal space [16], weaker binding affinity to LDL-C receptors [17], and longer plasma residence time [18]. Moreover, Srisawasdi et al. reported that the ratio of sdLDL-C/lbLDL-C was a potential biomarker for assessing lipid metabolic status in patients with metabolic syndrome [19]. Numerous epidemiologic studies and randomized clinical trials have suggested that elevated LDL-C is a major cause of CAD and the target to be controlled to reduce atherosclerotic cardiovascular disease risk [6, 20, 21]. However, a large proportion of atherosclerosis and CAD patients have normal range of blood LDL-C level. Recent large cohort studies have demonstrated that using a simple homogeneous sdLDL-C assay can predict the cardiovascular risk regardless of LDL-C level [15, 22–24]. These findings suggest that total LDL-C level cannot completely represent its biological effect and cannot fully represent blood lipid levels. Although many studies have shown that 10-year Framingham risk score and atherosclerotic CVD risk score were developed based on hypertension, LDL-C, HDL-C, TG, TC, age, smoking, and diabetes risk factors and used to predict CVD risk [25, 26], these prediction models have been reported that they have limitations to estimate future CVD risk [27, 28]. Hence, it is urgent to explore more potential risk factors. Therefore, more attention should be paid to explore the relationship between LDL-C subfractions and CAD risk, which may help to elucidate the differences among patients with CAD, and to establish the early warning to assess the risk of CAD.

In this study, we recruited 193 non-CAD controls and 2001 newly CAD patients including 1647 CAD patients who received lipid-lowering therapy and 354 who did not, collected their clinical features, and measured the concentration of blood lipid profile, including total cholesterol (TC), triglyceride (TG), high density lipoprotein cholesterol (HDL-C), LDL-C, and LDL-C subfractions. We aim to establish and evaluate six machine learning tools combining clinical features and blood lipid profile could predict the risk of CAD patients who did not receive lipid-lowering therapy or all CAD patients including 1647 CAD patients who received lipid-lowering therapy and 354 who did not.

## 2. Materials and Methods

### 2.1. Study Population

A total of 2001 newly diagnosed CAD consecutive patients including 1647 CAD patients who received lipid-lowering therapy and 354 who did not, as well as 193 non-CAD controls were recruited from the First People's Hospital of Pingdingshan and Luohe Central Hospital, from July 2018 to October 2019. The inclusion criteria for participants were as follows: (i) CAD patients were diagnosed by coronary angiography, which is defined as coronary artery stenosis ≥50% in at least one main vessel or its major branches as described [29]; (ii) the non-CAD controls were diagnosed by coronary angiography without any luminal stenosis or plaque in main vessels and branches; (iii) the age of all participates >18 years. The exclusion criteria were as follows: (i) patients who had prior CAD or revascularization (percutaneous or surgical) [30]; (ii) participants who do not understand this research study [3]; (iii) participants who had severe medical disease, such as liver or kidney disease, thyroid disease, and malignant diseases [29], as well as immune-related sickness, nephropathic diseases, and respiratory diseases and also physiological conditions related to immune responses such as pregnancy [31]. The exclusion criteria for the non-CAD controls were the same as what mentioned above. Clinical information of all participants was collected including age, gender, body mass index (BMI), smoking and drinking status, and other diseases history such as hypertension and diabetes mellitus.

Informed consent was obtained from each participant included in the study, and the study protocol conforms to the ethical guidelines of the latest version of Declaration of Helsinki, and the study protocol has been approved by Ethical Committee of the First People's Hospital of Pingdingshan and Luohe Central Hospital.

### 2.2. Blood Lipid Profile Detection

Blood samples were collected by serum separator tube and anticoagulant tube. Plasma was separated immediately after collection by 800 × g centrifugation for 10 min at 4°C. TC, TG, HDL-C, LDL-C, and glucose were measured in the Department of Clinical Laboratory. The reference ranges of TC, TG, HDL-C, and LDL-C were defined by 2016 Chinese guideline for the management of dyslipidemia in adults [32].

LDL-C subfractions were classified and quantified by LDL subfractions kit of Shanghai Biotecan Pharmaceuticals Co., Ltd. Briefly, the plasma mixed with liquid loading gel was added to the top of precast 3% polyacrylamide gel tubes. After 30 min of photopolymerization at room temperature, samples were electrophoresed in electrophoresis apparatus (Shanghai Biotecan Pharmaceuticals Co., Ltd.) for 70 min (3 mA/tub). Then, the densitometry was determined by Gel Scanner (Hunan Biotecan Medical Device Co., Ltd.). Last, LDL-C was separated into 7 subfractions (LDLC-1 to LDLC-7) as previously described [10].

### 2.3. Machine Learning Tools In Construction

In the present study, six machine learning tools were established to predict CAD risk, including k-nearest neighbor classifier (KNN) model, logistic regression (LR) model, support vector machine (SVM) model, decision tree (DT) model, multilayer perceptron (MLP) model, and extreme gradient boosting (XGBoost) model. Clinical characteristics and blood lipid profile are fit in the models. These models were tuned using a set of parameters, which were adjusted to obtain the average performance index. The tuning parameters of the six prediction models are listed for the optimization of the equations (Table S1). Python (version 3.8) was used as the basic language in the whole model, and NumPy, pandas, sklearn, XGBoost, and Matplotlib libraries were used to process the data and establish the models.

When to predict the CAD development risk in patients without lipid-lowering therapy, a total of 547 participants (193 non-CAD controls and 354 CAD patients) were randomly allocated into a training set (80%) and a test set (20%). When to predict the CAD development risk in all CAD patients, a total of 2194 participants (193 non-CAD controls and 2001 CAD patients) were randomly allocated into a training set (80%) and a test set (20%). In the training set, StratifiedKFold (*k* = 5) was used, and various parameter combinations were exhausted using grid search. For each model, the confusion matrix, area under the receiver operating characteristic (ROC) curve (AUC), accuracy, recall (sensitivity), precision, and F1 score were used to evaluate and compare the comprehensive performance of feature selection [33]. AUC is the main metric in evaluating binary classifiers and shows the true positive rate against the false positive rate [33]. Precision and recall are excellent metrics for capturing the aspects of model performance [34]. The *F*1 score takes the geometric mean of precision and recall [35]. In addition, the feature score (*F* score) rankings were measured by the total_gain metric in XGBoost model [3].

### 2.4. Statistical Analysis

Statistical analyses were performed using GraphPad Prism (version 6.0; GraphPad Software, Inc.) or SPSS 19.0 (IBM, NY, USA) and R 3.5.1 software. Categorical variables were presented by numbers or proportions, and differences in distribution between two groups were analyzed by chi-squared test. Continuous variables were presented using median with interquartile range (IQR) because they are non-Gaussian distributions data [36]. Nonparametric Mann–Whitney *U* test was used to analyze the difference between two groups or Kruskal-Wallis *H* test followed by Dunn's post hoc test that was used to analyze the difference among three groups. Correlation analysis among CAD development, clinical features, and blood lipid profile was conducted by the Pearson correlation method. A *P* < 0.05 was considered statistically significant.

## 3. Results

### 3.1. Comparison of Clinical Characteristics between the Non-CAD Controls and CAD Patients

The clinical characteristics of all participants (193 non-CAD controls and 2001 CAD patients) were collected in Table 1. The CAD patients were older than the non-CAD controls. The median BMI of CAD patients was significantly higher than that of the non-CAD controls. Moreover, a noticeably higher prevalence of drinking, hypertension, and diabetes mellitus was observed in CAD patients, comparing to non-CAD controls (Table 1).

### 3.2. Comparison of the Blood Lipid Profile Levels among the Non-CAD Controls, CAD Patients Who Received Lipid-Lowering Therapy, and those Who Did Not

**Figure 1:**
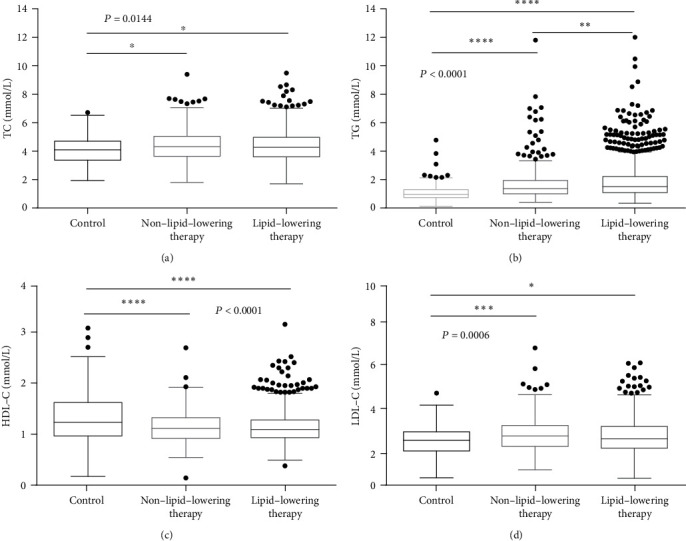
Comparison of blood lipids level among non-CAD controls and CAD patients who received lipid-lowering therapy or those who did not. The different expressions of (a) TC, (b) TG, (c) HDL-C, and (d) LDL-C among three groups. ^∗^*p* < 0.05, ^∗∗^*p* < 0.01, ^∗∗∗^*p* < 0.001, and ^∗∗∗∗^*p* < 0.0001.

We detected the levels of TC, TG, HDL-C, and LDL-C and compared among 193 non-CAD controls (control group), 1647 CAD patients who received lipid-lowering therapy (lipid-lowering therapy group), and 354 CAD patients did not receive any lipid-lowering therapy (non-lipid-lowering therapy group). The level of TC is significantly higher in the two groups of CAD patients than that in the control group. The level of TG is the highest in the lipid-lowering therapy group than that in the other two groups (Figures 1(a) and 1(b)). Moreover, HDL-C is the highest in the control group than in the two groups of CAD patients, while LDL-C was the lowest in the control group (Figures 1(c) and 1(d)). However, there are no HDL-C and LDL-C differences between the two groups of CAD patients.

**Figure 2:**
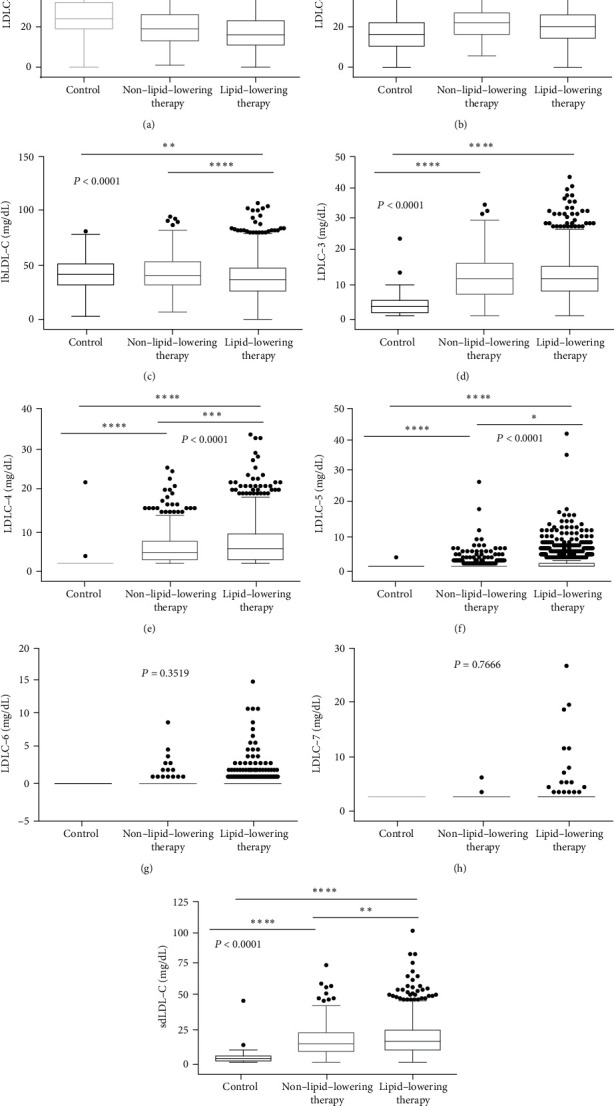
Comparison of LDL-C subfractions levels among non-CAD controls and CAD patients who received lipid-lowering therapy or those who did not. The different expressions of (a) LDLC-1, (b) LDLC-2, (c) lbLDL-C, (d) LDLC-3, (e) LDLC-4, (f) LDLC-5, (g) LDLC-6, (h) LDLC-7, and (i) sdLDL-C among three groups. ^∗^*p* < 0.05, ^∗∗^*p* < 0.01, ^∗∗∗^*p* < 0.001, and ^∗∗∗∗^*p* < 0.0001.

In addition, we also compared the levels of LDL-C subtractions (LDLC-1 to LDLC-7) among the three groups. The level of LDLC-1 was significantly lower, while LDLC-2 was noticeably higher in the two groups of CAD patients than that in the control group. The total lbLDL-C level was only higher in the control group than in the other two groups (Figures 2(a)–2(c)). The concentrations of LDLC-3, LDLC-4, LDLC-5, and sdLDL-C are significantly higher in both two groups of CAD patients than that in the control group. Moreover, the concentrations of LDLC-4, LDLC-5, and sdLDL-C are significantly higher in the lipid-lowering therapy than that in the non-lipid-lowering therapy group. However, both LDLC-6 and LDLC-7 showed no significant differences among the three groups (Figures 2(d)–2(i)).

### 3.3. The Abnormal Rates of Blood Lipid Profile among in the Non-CAD Controls and the CAD Patients Who Received Lipid-Lowering Therapy and those Who Did Not

Besides that, we found that the abnormal rates of TC, TG, HDL-C, and LDL-C were 11.92%, 15.03%, 27.98%, and 4.66% in the control group; 20.34%, 37.57%, 34.46%, and 15.25% in the non-lipid-lowering therapy group; 19.31%, 47.42%, 35.03%, and 12.26% in the lipid-lowering therapy group (Table 2), respectively. Moreover, we further investigated the abnormal rates of LDL-C subfractions separately. Surprisingly, the abnormal rates of LDLC-3, LDLC-4, and LDLC-5 were 80.79%, 76.27%, and 24.29% in the non-lipid-lowering therapy group and 84.52%, 84.46%, and 32.67% in the lipid-lowering therapy group, respectively. On the contrary, the abnormal rates of LDLC-3 (3.11%), LDLC-4 (1.04%), and LDLC-5 (0.52%) were very low in the control group (Table 2).

### 3.4. Comparison of the sdLDL-C Subfractions among the Non-CAD Controls, the CAD Patients Who Received Lipid-Lowering Therapy, and those Who Did Not

According to the above results, we found the very high abnormal rates of LDLC-3 and LDLC-4 in the lipid-lowering therapy and non-lipid-lowering therapy groups. However, it remains unknown that whether the abnormal rates of LDLC-3 and LDLC-4 in CAD patients with normal LDL levels are high or not. Thus, we divided LDL-C normal and LDL-C high subgroups among the three groups separately and detected the abnormal rates of sbLDL-C subtractions in the two subgroups. Surprisingly, the abnormal rates of LDLC-3 and LDLC-4 were still high in all CAD patients with normal LDL-C levels. To be specific, the abnormal rates of LDLC-3 and LDLC-4 were 82.98% and 84.43%, respectively, in the lipid-lowering therapy group, while the abnormal rates of LDLC-3 and LDLC-4 were 78.33% and 75.67%, respectively, in the non-lipid-lowering therapy group. However, the abnormal rates of these two LDL-C subfractions remained low in the non-CAD controls with normal LDL-C levels (Table 3). These results indicated that LDLC-3 and LDLC-4 were the main components in the sbLDL-C subtractions and may play an important role in CAD development.

### 3.5. Correlation Analysis among CAD Risk, Clinical Characteristics, and Blood Lipid Profile

**Figure 3:**
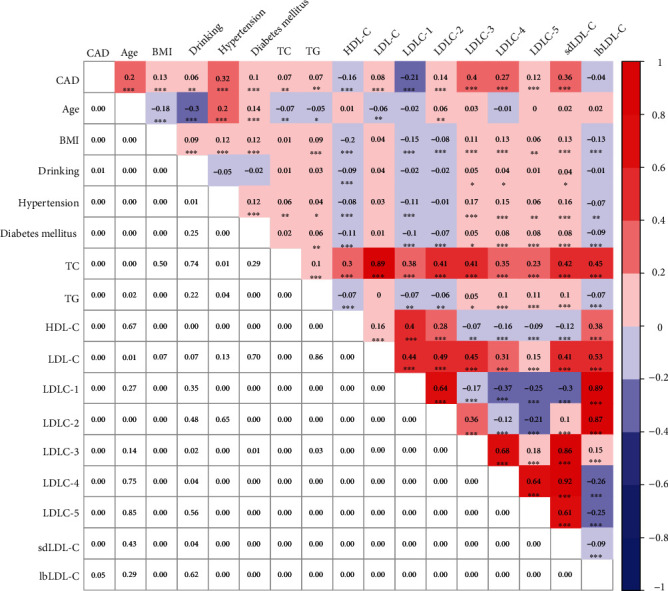
Correlation analysis among clinical characteristics, blood lipid profile, and CAD. The value in grids is Pearson correlation coefficient (Pearson's *r*), which is also marked by colors. ^∗^*p* < 0.05, ^∗∗^*p* < 0.01, and ^∗∗∗^*p* < 0.001.

In order to analyze the correlation among clinical data, blood lipid profile, and CAD risk, Pearson correlation analysis was employed to evaluate the correlation among them (Figure 3). Since sex and smoking had no significant differences between the non-CAD controls and CAD patients (Table 1), and both LDLC-6 and LDLC-7 were not noticeably expressed among the three groups (Figures 2(g) and 2(h)), sex, smoking, LDLC-6, and LDLC-7 were excluded in the Pearson correlation analysis. We found that age (*r* = 0.2, *p* < 0.001), hypertension (*r* = 0.32, *p* < 0.001), LDLC-3 (*r* = 0.4, *p* < 0.001), LDLC-4 (*r* = 0.27, *p* < 0.001), and sdLDL-C (*r* = 0.36, *p* < 0.001) were significantly positively correlated with CAD, while LDLC-1 (*r* = −0.21, *p* < 0.001) was significantly negatively correlated with CAD. Moreover, TC was significantly positively correlated with HDL-C (r = 0.3, p <0.001), LDL-C (r = 0.89, *p* < 0.001), LDLC-1 (*r* = 0.38, *p* < 0.001), LDLC-2 (*r* = 0.41, *p* < 0.001), LDLC-3 (*r* = 0.41, *p* < 0.001), LDLC-4 (*r* = 0.35, *p* < 0.001), sdLDL-C (*r* = 0.42, *p* < 0.001), and lbLDL-C (*r* = 0.38, *p* < 0.001). HDL-C had strongly positive correlation with LDLC-1 (*r* = 0.4, *p* < 0.001), LDLC-2 (*r* = 0.28, *p* < 0.001), and lbLDL-C (*r* = 0.38, *p* < 0.001). Meanwhile, LDL-C had significantly positive correlation with LDLC-1 (*r* = 0.44, *p* < 0.001), LDLC-2 (*r* = 0.49, *p* < 0.001), LDLC-3 (*r* = 0.45, *p* < 0.001), and LDLC-4 (*r* = 0.31, *p* < 0.001), as well as sdLDL-C (*r* = 0.451, *p* < 0.001) and lbLDL-C (*r* = 0.53, *p* < 0.001). In addition, lbLDL-C had strongly positive correlation with LDLC-1 (*r* = 0.89, *p* < 0.001) and LDLC-2 (*r* = 0.87, *p* < 0.001), while lbLDL-C was significantly negatively correlated with LDLC-4 (*r* = −0.26, *p* < 0.001) and LDLC-5 (*r* = −0.25, *p* < 0.001). Importantly, SdLDL-C had strongly positive correlation with LDLC-3 (*r* = 0.86, *p* < 0.001), LDLC-4 (*r* = 0.92, *p* < 0.001), and LDLC-5 (*r* = 0.61, *p* < 0.001), while sdLDL-C was significantly negatively correlated with LDLC-1 (*r* = −0.3, *p* < 0.001) (Figure 3).

### 3.6. Establish and Compare Six Machine Learning Models to Predict CAD Development

**Figure 4:**
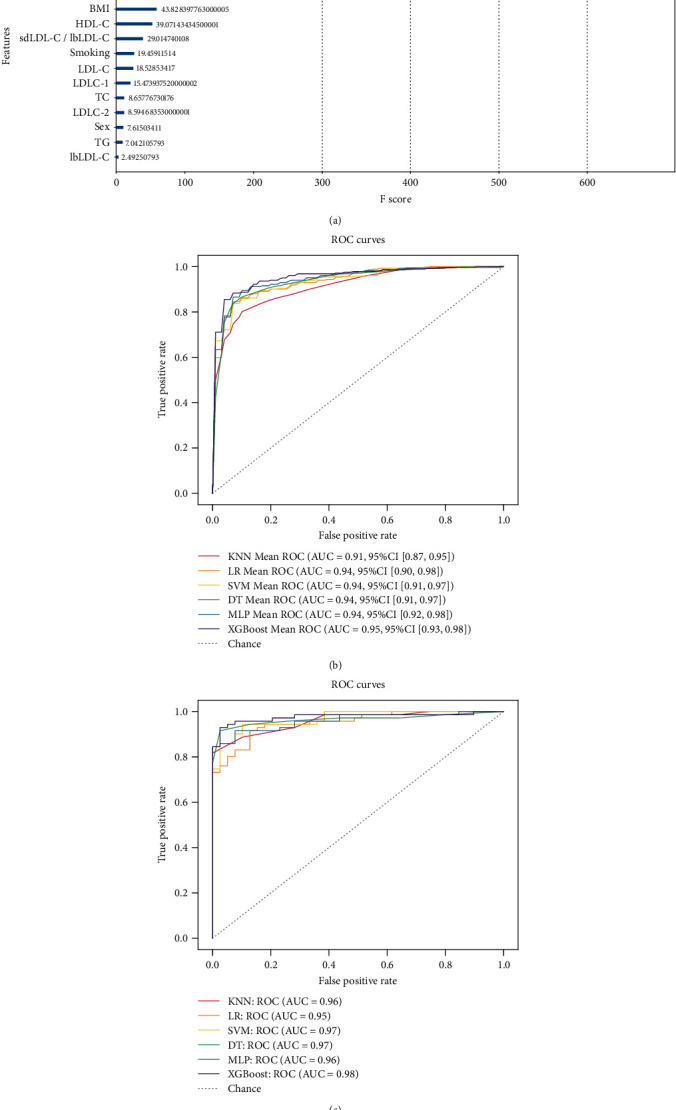
Six models were established to predict the development of risk CAD patients who did not receive any lipid-lowering treatment. The figure shows the average ROC curves of the 6 models in the training set and test set. (a) Analysis of the importance of each feature including clinical data and lipid profile in the XGBoost model. The relative importance is quantified by assigning a weight between 0 and 600 for each variable. (b) Mean AUC values and 95% CIs of all models are shown in the training set. (c) The AUC values of all models are shown in the test set. ROC: receiver operating characteristic; AUC: area under the ROC curve; CI: confidence interval; KNN: k-nearest neighbors; LR: logistic regression; SVM: support vector machine; DT: decision tree; MLP: multilayer perceptron; XGBoost: extreme gradient boosting.

On the one hand, in order to predict the CAD risk of patients who did not receive lipid-lowering therapy, we firstly used XGBoost model to analyze the importance of features including 7 clinical features (age, sex, BMI, smoking, drinking, hypertension, and diabetes mellitus) and 14 blood lipid profile (TC, TG, HDL-C, LDL-C, sdLDL-C, lbLDL-C, LDLC-1 to LDLC-7, and sdLDL-C/lbLDL-C), and the feature score (*F* score) rankings were measured by the total_gain metric in XGBoost. However, only 16 factors (age, sex, BMI, smoking, drinking, hypertension, TC, TG, HDL-C, LDL-C, sdLDL-C, lbLDL-C, LDLC-1, LDLC-2, LDLC-4, and sdLDL-C/lbLDL-C) were obtained in the rankings (Figure 4(a)). Among them, sdLDL-C, LDLC-4, and hypertension ranked top 3 in the feature importance rankings, while sex, TG, and lbLDL-C ranked lower (Figure 4(a)). Therefore, these 16 factors were enrolled into the six machine learning tools.

After that, a total of 547 participants (193 non-CAD controls and 354 CAD patients without receiving lipid-lowering therapy) were randomly allocated into a training set (80%) and a test set (20%). StratifiedKFold (*k* = 5) was used in the training set. After fitting in the training set, each model is evaluated by the test set. For each model, the evaluation indicators used were the confusion matrix, AUC, recall (sensitivity), precision, accuracy, and F1 score. The ROC curve is widely used to validate the performance of prediction models, and the average AUC and 95% CI are shown in Figures 4(b) and 4(c). In the training set, all models had AUC values above 0.90 (Figure 4(b)), and the accuracy, precision, recall, and F1 score were above 0.83, 0.87, 0.85, and 0.86, respectively (Table 4). Among them, XGBoost model had the highest AUC (0.95), as wells as the highest accuracy (0.90), precision (0.94), and F1 score (0.92). In addition, the MLP model also obtained the same highest *F*1 score (0.92) and the highest recall (0.91). Importantly, in the test set, all models obtained the AUC values above 0.94 (Figure 4(c)), and the accuracy, precision, recall, and F1 score were above 0.84, 0.85, 0.92 and 0.88, respectively (Table 5). Interestingly, XGBoost still obtained the highest AUC (0.98), accuracy (0.93), precision (0.93), recall (0.96), and F1 score (0.94). Meanwhile, the DT model obtained the same highest precision (0.93) and *F*1 score (0.94).

**Figure 5:**
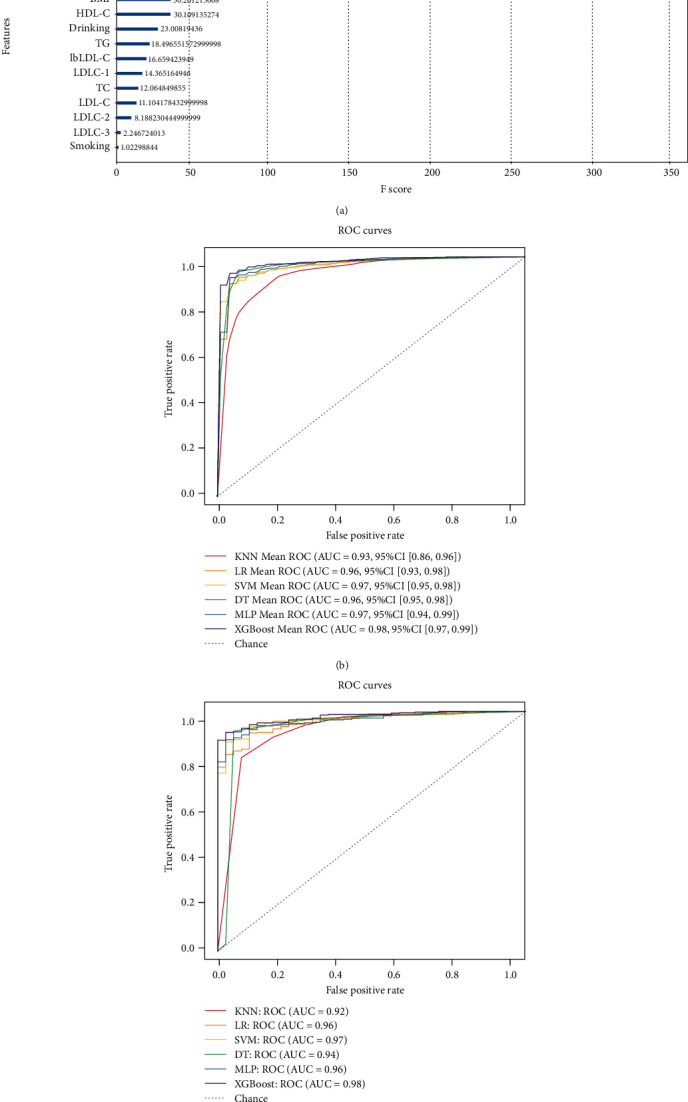
Six models were established to predict the development risk of all CAD patients. The figure shows the average ROC curves of the 6 models in the training set and test set. (a) Analysis of the importance of each feature including clinical data and lipid profile in the XGBoost model. The relative importance is quantified by assigning a weight between 0 and 350 for each variable. (b) Mean AUC values and 95% CIs of all models are shown in the training set. (c) The AUC values of all models are shown in the test set. ROC: receiver operating characteristic; AUC: area under the ROC curve; CI: confidence interval; KNN: k-nearest neighbors; LR: logistic regression; SVM: support vector machine; DT: decision tree; MLP: multilayer perceptron; XGBoost: extreme gradient boosting.

On the other hand, in order to verify whether the six machine learning tools combing clinical features and blood lipid profile could predict the risk of all CAD patients including 1647 CAD patients who received lipid-lowering therapy and 354 who did not, we enrolled all CAD patients in the six predictive models. We also used the XGBoost model to analyze the importance of 21 features as above mentioned. Finally, only 16 factors obtained the importance ranking including age, BMI, smoking, drinking, hypertension, TC, TG, HDL-C, LDL-C, sdLDL-C, lbLDL-C, LDLC-1, LDLC-2, LDLC-3, LDLC-4, and sdLDL-C/lbLDL-C. Interestingly, sdLDL-C, LDLC-4, and sdLDL-C/lbLDL-C ranked top 3 in the feature importance rankings, while LDLC-2, LDLC-3, and smoking ranked lower (Figure 5(a)). Thus, these 16 factors were enrolled into the six machine learning tools.

After that, a total of 2194 participants (193 non-CAD controls and 2001 CAD patients) were randomly allocated into a training set (80%) and a test set (20%). In the training set, all models obtained AUC values above 0.92 (Figure 5(b)), and the accuracy, precision, recall, and F1 score were above 0.89, 0.94, 0.89, and 0.93, respectively (Table 6). Among them, XGBoost model had the highest AUC (0.98), as well as the highest accuracy (0.95), recall (0.98), and *F*1 score (0.97). Furthermore, the LR, SVM, and DT model obtained the same highest precision (0.99). MLP showed the same highest accuracy (0.95) and *F*1 score (0.97). Importantly, in the test set, all models obtained the AUC values above 0.91 (Figure 5(c)), and the accuracy, precision, recall, and F1 score were above 0.87, 0.94, 0.87, and 0.92, respectively (Table 7). Interestingly, XGBoost still obtained the highest AUC (0.98), accuracy (0.95), recall (0.98), and *F*1 score (0.97). Moreover, KNN and XGBoost obtained the same highest *F*1 score (0.97) and recall (0.98). LR, SVM, and DT models all obtained the same highest precision (0.99). The results indicated that machine learning tools combing clinical features and blood lipid profile showed excellent performance to predict the CAD risk.

## 4. Discussion

In the past decades, a large number of studies have already reported many possible CAD risk factors, such as BMI [37], HDL-C, LDL-C, TG and TC [38], smoking, diabetes, and hypertension [39], in order to early assess the risk of CAD. Thus, in this study, we recruited 193 non-CAD controls and 2001 CAD patients and collected related risk clinical data to find out the potential risk factors for CAD. It has been reported that obesity is a common cause of cardiovascular deaths in the developed countries [40]. Moreover, diabetes has been observed to be associated with hyperlipidemia, which is characterized by increased levels of TC and decreased levels of HDL-C [41]. It has been observed that diabetic patients have higher risk of suffering from CAD than nondiabetic people [42]. Besides that, hypertension has also been frequently associated with metabolic disorders like insulin resistance or dyslipidemia, which are also known to be the risk factors of CAD [43]. Similar to the above-mentioned studies, in this study, we also found that a significantly higher prevalence of BMI, hypertension, and diabetes mellitus was observed in the CAD patients.

Besides that, increasing evidence suggests that inflammation plays an important role in the pathogenesis of CAD [44, 45]. Transforming growth factor-*β*1 (TGF-*β*1) is a multifunctional cytokine that regulates cell growth, differentiation, and matrix production and has a pivotal role in wound healing [46]. The high expression of TGF-*β*1 level was observed during the development of many human diseases, such as periodontal disease [47] and CAD [48]. Matarese et al. reported that both TGF-*β*1 and vascular endothelial growth factor (VEGF) played an important regulating role in the orchestration of the immune response in periodontal disease [47]. Although the serum level of total TGF-*β*1 was upregulated in the CAD patients than in the control samples, Wang and Zhang have shown that the AUC of serum levels of total TGF-*β*1 in the diagnosis of CAD was only 0.5109 [49]. Moreover, expression of VEGF is upregulated by hypoxia, inflammation, wound-healing, and other pathological processes [50]. A previous study showed that circulating levels of total VEGF-A and VEGF-A165b in CAD patients were associated with syntax score, indicating the severity and complexity of CAD [51]. Moreover, transglutaminase 2 (TG2), a protein cross-linking enzyme according to Matarese and Curro, has showed a positive correlation between TG2 and RANKL/OPG mRNA ratio, suggesting that TG2 may be involved in molecular mechanisms of inflammatory response occurring in periodontal disease [52]. In the acute myocardial infarction (AMI), model study showed that TGF-*β*1-induced transition of cardiofibroblasts into myofibroblast-like cells can be attenuated by the TG2 inhibitor 1–155, suggesting a new role for TG2 in regulating TGF-*β*1 signaling in addition to its role in latent TGF-*β*1 activation [53]. Besides that, cholesterol-induced sterile inflammation is thought to be central to this process via activation of a protein complex called the nucleotide-binding oligomerization domain-, leucine-rich repeat-, and pyrin domain-containing 3 (NLRP3) inflammasome. The comorbidity of smoking, hypertension, diabetes, elevated LDL-C and lipoprotein(a), or decreased HDL-C also correlated with increased NLRP3 protein expression in the aorta [54]. Zheng and Xing found that coronary atherosclerosis patients expressed high levels of NLRP3 in the aorta, which was correlated to heart disease severity [55]. However, no study has investigated whether combing these above-mentioned inflammatory factors could predict CAD risk. Hence, further researches are required to establish prediction models combing inflammatory factors to predict CAD risk in the near future. Interestingly, in recent years, the gut microbiota has been shown the capacity to contribute to substantial variation in blood lipid composition and cause CAD development [56], which can be detected by metagenomics and 16 s DNA sequencing approaches [57]. Correlations have been shown between CAD and the gut microbiota; however, the potential causal relationships are much more complex and challenging to demonstrate in the near future. In addition, we detected the traditional blood lipids in all participates and found that LDL-C level was significantly higher in the CAD patients. Large numbers of previous studies have demonstrated that LDL-C plays a crucial role in the pathogenesis and the development of CVD [58, 59]. However, CAD occurred in many patients with low LDL-C levels, and cardiovascular events even still appeared in CAD patients with intensive lipid-lowering therapy [9, 60]. Therefore, we further detected the concentrations of LDL-C subfractions by Lipoprint LDL System. Surprisingly, we found that the levels of LDLC-3 and LDL-4 were significantly higher in the CAD patients than that in the non-CAD controls. Moreover, the very high abnormal rates of LDLC-3 and LDLC-4 were found in the CAD patients, even in the normal LDL-C subgroup of CAD patients. However, they are very low in the non-CAD controls. These results indicated that specific LDL-C subfractions showed powerful potential to screen high-risk CAD patients whose LDL-C levels were in normal range. Meanwhile, it can explain why many studies have reported that people with abnormal levels of sdLDL-C have high risk of cardiovascular and cerebrovascular events, even if their LDL-C levels are in normal range [61, 62]. Besides that, we found that sdLDL-C was significantly positively correlated with CAD, which is consistent with many prospective observational studies that reported sdLDL-C level was positive association with CAD [15, 23, 63]. Meanwhile, we found that LDLC-3 and LDLC-4 were noticeably correlated with CAD too. The results suggest that LDLC-3 and LDL-4 make the great contribution for sdLDL-C composition and might be the main cause of CAD risk. However, further research studies are required to elucidate the mechanisms in the near feature.

With the development of artificial intelligence (AI), machine learning is a branch of AI that describes computer models learning how to do tasks on the basis of source data rather than being rigidly programmed to do them [64, 65]. It has been attracted substantial attention for its applications in disease diagnosis, prognosis, and treatment [34]. Machine learning has been increasingly applied in the cardiovascular research field. For example, Al'Aref and Maliakal have shown that using XGBoost model combined with coronary artery calcium scoring, age, sex, symptoms, and cardiovascular risk factors can predict obstructive CAD and yield a good AUC of 0.881 [30]. Gupta and Slater have conducted a research study that used LR, SVM, artificial neural network, and Bayesian network models combing 59 variables from real-world observational data set of 303 Iranian patients at risk for CAD. The results indicated that four models showed all AUC above 0.90 for predicting CAD [66]. However, in the above-mentioned machine learning studies for predicting CAD risk, they did not enroll sdLDL-C subfractions features. In the present study, we use six machine learning tools including KNN, LR, DT, SVM, MLP, and XGBoost-combined clinical features and blood lipid profile including sdLDL-C subfractions to predict CAD risk. We found that all models performed well in the prediction of CAD risk, which is consistent with a previous study that using above-mentioned models combing clinical data and sdLDL-C subfractions showed good performance for predicting CAD risk [3]. In addition, SVM, KNN, LR, and XGBoost models have also been reported to predict chronic kidney disease [67] and chronic obstructive pulmonary disease in Chinese population [33]. Importantly, we found that sdLDL-C ranked top 1 and LDLC-4 ranked top 2 in the feature rankings, while the study by Wu and Yang [3] indicated that sdLDL-4 ranked top 1 in the feature rankings, but sdLDL-C seems less important in that model. Therefore, large samples are needed to verify this issue in the near future. Surprisingly, although the very high abnormal rate of LDLC-3 was found in the CAD patients, it is less important in the models in our study. Interestingly, hypertension ranked top 5 in all features but ranked top 1 in the 7 clinical data. Hypertension is a well-known independent risk factor for CAD, and patients with hypertension often accompanied with abnormal lipid metabolism, which could significantly increase the risk of cardiovascular events [68, 69], which may explain why hypertension ranked higher in the model. LDL-C level cannot reflect the risk of CAD and that may explain why LDL-C ranked lower in the rankings. Meanwhile, the results indicated that sex, smoking, TC, TG, lbLDL-C as well as LDLC-1, and LDLC-2 were less important in the model.

There were some limitations in this study. Firstly, the non-CAD sample size used was relatively small, and the total CAD patients and non-CAD controls was unbalanced. Secondly, we did not assess the inflammatory factors associated with CAD and input the prediction models to predict the CAD risk. Thus, further studies need to be established prediction combing-related inflammatory factors to predict CAD risk. Thirdly, these findings should be validated in a larger cohort in multicenter before these models can be applied in the clinic for CAD prediction.

In conclusion, we demonstrated that LDLC-3 and LDLC-4 were the main components of sdLDL-C and may be the main risk for CAD development. Moreover, LDLC-3, LDLC-4, and sdLDL-C were significantly positively correlated with CAD. Importantly, we identified that both sdLDL-C and LDLC-4 play important roles in the prediction models rather than LDL-C. In addition, this study also revealed that six machine learning tools combined with clinical features and lipid profile showed excellent overall predictive power and could potentially be beneficial for the early prediction of the risk of CAD in the Chinese population.

## Figures and Tables

**Table 1:** The clinical characteristics of 193 non-CAD controls and 2001 CAD patients.

Characteristics	Non-CAD controls (*n* = 193)	CAD patients (*n* = 2001)	*p* value
Age (years) (Median with IRQ)	52 (43-68)	64 (54-72.5)	<0.0001
Gender			0.51
(i) Male	99	1076	
(ii) Female	94	925	
BMI (kg/m^2^) (median with IRQ)	22.86 (20.83-26.04)	24.69 (23.40-27.24)	<0.0001
Smoking			0.065
(i) Yes	24	354	
(ii) No	169	1647	
Drinking			0.008
(i) Yes	20	358	
(ii) No	173	1643	
Hypertension			<0.0001
(i) Yes	43	1481	
(ii) No	150	520	
Diabetes mellitus			<0.0001
(i) Yes	43	775	
(ii) No	150	1226	
Lipid-lowering therapy			/
(i) Yes	0	1647	
(ii) No	193	354	

CAD: coronary artery disease; IRQ: interquartile range; BMI: body mass index; ∗*p* < 0.05 was considered to be significant.

**Table 2:** The abnormal rate of blood lipid levels among the non-CAD controls, the CAD patients who received lipid-lowering therapy, and those who did not.

Variables	Threshold	Control group (*n* = 193)	Non-lipid-lowering therapy group (*n* = 354)	Lipid-lowering therapy group (*n* = 1647)
		Abnormal rate	Abnormal rate	Abnormal rate
TC (mmol/L)	≥5.20	11.92% (23/193)	20.34% (72/354)	19.31% (318/1647)
TG (mmol/L)	≥1.7	15.03% (29/193)	37.57% (133/354)	47.42% (781/1647)
HDL-C (mmol/L)	<1	27.98% (54/193)	34.46% (122/354)	35.03% (577/1647)
LDL-C (mmol/L)	≥3.4	4.66% (9/193)	15.25% (54/354)	12.26% (202/1647)
LDLC-1 (mg/dL)	/	/	/	/
LDLC-2 (mg/dL)	/	/	/	/
LDLC-3 (mg/dL)	>6	3.11% (6/193)	80.79% (286/354)	84.52% (1392/1647)
LDLC-4 (mg/dL)	>0	1.04% (2/193)	76.27% (270/354)	84.46% (1391/1647)
LDLC-5 (mg/dL)	>0	0.52% (1/193)	24.29% (86/354)	32.67% (538/1647)
LDLC-6 (mg/dL)	>0	0.00% (0/193)	4.24% (15/354)	5.34% (88/1647)
LDLC-7 (mg/dL)	>0	0.00% (0/193)	0.56% (2/354)	1.09% (18/1647)
lbLDL-C (mg/dL)	/	/	/	/
sdLDL-C (mg/dL)	/	/	/	/
sdLDL-C/lbLDL-C	/	/	/	/

CAD: coronary artery disease; TC: total cholesterol; TG: triglyceride; HDL-C: high density lipoprotein cholesterol; LDL-C: low density lipoprotein cholesterol; sdLDL-C: small dense LDL-C; lbLDL-C: large buoyant LDL-C.

**Table 3:** The abnormal rate of sdLDL-C subfractions in the CAD patients and non-CAD controls who were classified into LDL-C normal and high subgroups.

sdLDL-C subfractions	Control group(*n* = 193)		Non-lipid-lowering therapy group (*n* = 354)		Lipid-lowering therapy group (*n* = 1647)	
	LDL-C normal subgroup (*n* = 184)	LDL-C high subgroup (*n* = 9)	LDL-C normal subgroup (*n* = 300)	LDL-C high subgroup (*n* = 54)	LDL-C normal subgroup (*n* = 1445)	LDL-C high subgroup (*n* = 202)
	Abnormal rate	Abnormal rate	Abnormal rate	Abnormal rate	Abnormal rate	Abnormal rate
LDLC-3 (>6 mmol/L)	3.26% (6/184)	0%	78.33% (235/300)	92.59% (50/54)	82.98% (1199/1445)	95.54% (193/202)
LDLC-4 (>0 mmol/L)	1.09% (2/184)	0%	75.67% (227/300)	77.78% (42/54)	84.43% (1220/1445)	84.65% (171/202)
LDLC-5 (>0 mmol/L)	0.54% (1/184)	0%	23.33% (70/300)	29.63% (16/54)	31.35% (453/1445)	42.08% (85/202)
LDLC-6 (>0 mmol/L)	0% (0/184)	0%	4.00% (12/300)	5.56% (3/54)	5.33% (77/1445)	5.54% (11/202)
LDLC-7 (>0 mmol/L)	0% (0/184)	0%	0.67% (2/300)	0% (0/54)	1.04% (15/1445)	1.49% (3/202)

CAD: coronary artery disease; sdLDL-C: small dense LDL-C. LDL-C, low density lipoprotein cholesterol.

**Table 4:** The performance of six models in the prediction of 354 CAD patients without lipid-lowering therapy in the training set.

Metrics	KNN (95% CI)	LR (95% CI)	SVM (95% CI)	DT (95% CI)	MLP (95% CI)	XGBoost (95% CI)
Accuracy	0.84 (0.79-0.88)	0.86 (0.82-0.93)	0.86 (0.83-0.89)	0.86 (0.82-0.88)	0.89 (0.85-0.93)	0.90 (0.87-0.94)
Precision	0.88 (0.84-0.91)	0.90 (0.85-0.95)	0.92 (0.90-0.94)	0.92 (0.83-0.99)	0.93 (0.91-0.94)	0.94 (0.92-0.98)
Recall	0.87 (0.80-0.91)	0.90 (0.86-0.94)	0.86 (0.823-0.88)	0.87 (0.79-0.93)	0.91 (0.86-0.96)	0.90 (0.86-0.96)
*F*1 score	0.87 (0.83-0.91)	0.90 (0.86-0.95)	0.89 (0.86-0.91)	0.89 (0.87-0.91)	0.92 (0.88-0.95)	0.92 (0.89-0.95)

KNN: k-nearest neighbors; LR: logistic regression; SVM: support vector machine; DT: decision tree; MLP: multilayer perceptron; XGBoost: extreme gradient boosting; 95% CI: 95% confidence interval.

**Table 5:** The performance of six models in the prediction of 354 CAD patients without lipid-lowering therapy in the test set.

Metrics	KNN	LR	SVM	DT	MLP	XGBoost
Accuracy	0.85	0.89	0.91	0.92	0.86	0.93
Precision	0.86	0.89	0.92	0.93	0.87	0.93
Recall	0.93	0.94	0.94	0.94	0.93	0.96
*F*1 score	0.89	0.92	0.93	0.94	0.9	0.94

KNN: k-nearest neighbors; LR: logistic regression; SVM: support vector machine; DT: decision tree; MLP: multilayer perceptron; XGBoost: extreme gradient boosting.

**Table 6:** The performance of six models in the prediction of all CAD patients in the training set.

Metrics	KNN (95% CI)	LR (95% CI)	SVM (95% CI)	DT (95% CI)	MLP (95% CI)	XGBoost (95% CI)
Accuracy	0.94 (0.93-0.95)	0.90 (0.86-0.93)	0.91 (0.89-0.93)	0.92 (0.91-0.94)	0.95 (0.94-0.96)	0.95 (0.93-0.96)
Precision	0.95 (0.94-0.95)	0.99 (0.99-1.00)	0.99 (0.99-1.00)	0.99 (0.99-1.00)	0.97 (0.96-0.98)	0.97 (0.96-0.98)
Recall	0.99 (0.98-1.00)	0.90 (0.86-0.93)	0.91 (0.90-0.93)	0.92 (0.90-0.93)	0.97 (0.96-0.98)	0.98 (0.97-0.99)
*F*1 score	0.97 (0.96-0.97)	0.94 (0.92-0.96)	0.95 (0.94-0.96)	0.96 (0.95-0.96)	0.97 (0.96-0.98)	0.97 (0.97-0.98)

KNN: k-nearest neighbors; LR: logistic regression; SVM: support vector machine; DT: decision tree; MLP: multilayer perceptron; XGBoost: extreme gradient boosting; 95% CI: 95% confidence interval.

**Table 7:** The performance of six models in the prediction of all CAD patients in the test set.

Metrics	KNN	LR	SVM	DT	MLP	XGBoost
Accuracy	0.94	0.88	0.90	0.92	0.94	0.95
Precision	0.95	0.99	0.99	0.99	0.97	0.97
Recall	0.98	0.88	0.9	0.91	0.96	0.98
*F*1 score	0.97	0.93	0.94	0.95	0.96	0.97

KNN: k-nearest neighbors; LR: logistic regression; SVM: support vector machine; DT: decision tree; MLP: multilayer perceptron; XGBoost: extreme gradient boosting.

## Data Availability

The original contributions presented in the study are included in the article, and further inquiries can be directed to the corresponding authors.

## Supplementary Materials

Supplementary Materials Table S1: tuning parameters in the six predictive models.
