# Supplementary material for: Development of Machine Learning Tools for Predicting Coronary Artery Disease in the Chinese Population
Source: Dis Markers. 2022 Nov 17;2022:6030254. doi: 10.1155/2022/6030254 (PMC9691305; doi:10.1155/2022/6030254)
Supplement: Supplementary Materials — Table S1: tuning parameters in the six predictive models. [file 6030254.f1.docx]

Table S1: Tuning parameters in the six predictive models.

|  | | |
| --- | --- | --- |
|  | Tuning parameters | |
| Models | For predicting the risk of CAD patients without lipid-lowering therapy | For predicting the risk of all CAD patients |
| SVM | C =1 | C =1 |
|  | gamma =“auto” | gamma =“auto” |
|  |  |  |
| MLP | hidden_layer_sizes =(40, 40) | hidden_layer_sizes =(25,) |
|  | activation=“logistic” | activation =“tanh” |
|  | learning_rate=“constant” | learning_rate =“constant” |
|  |  |  |
| XGBoost | max_depth =2 | max_depth =2 |
|  | n_estimators =100 | n_estimators =100 |
|  | learing_rate =0.15 | learing_rate=0.15 |
|  |  |  |
| KNN | n_neighbors=9 | n_neighbors =10 |
|  |  |  |
| DT | Splitter = “best” | Splitter = “best” |
|  | Criterion =“gini” | Criterion =“gini” |
|  | max_depth =4 | max_depth=4 |
|  | min_samples_leaf =26 | min_samples_leaf=11 |
|  |  |  |
| LR | C =0.08 | C=0.08 |
|  | max_iter =200 | max_iter=200 |

SVM, support vector machine; MLP, multilayer perceptron; XGBoost, extreme gradient boosting; KNN, k-nearest neighbors; DT, decision tree; LR, logistic regression.
